# Our New York Letter

**Published:** 1888-02

**Authors:** 


### Correspondence.

### OUR NEW YORK LETTER.

OPENING OF SLOANE MATERNITY HOSPITAL AND VANDERBILT CLINIC—ADDRESS BY DR. T. GAILLARD THOMAS—SEPTIC PERITONITIS IN THE NEW-BORN—THE NEW YORK BURIAL REFORM ASSOCIATION—PROGRESS OF CREMATION.

*Editors Atlanta Medical and Surgical Journal:*

One of the most important medical events of the winter has been the opening of the Sloane Maternity Hospital and the Vanderbilt Clinic. The ceremonies were held in the lower hall of the College of Physicians and Surgeons, which adjoins the two new structures, and although the weather was very unfavorable, a large audience was present. Among the most noticeable gentlemen upon the ptatform were Cornelius and Fred. W. Vanderbilt, Drs. L. A. Sayre, Fordyce Barker, F. N. Otis, H. B. Sands, C. R. Agnew, A. Jacobi and Thomas M. Markoe. The exercises began with prayer by Rev. Dr. John Hall, after which Dr. Dalton, the President, introduced Dr. T. Gaillard Thomas as the orator of the occasion. Dr. Thomas began his address with the following words, "*Vita brevis—ars longa.*" Among the arts, he said, there were none which had undergone more rapid changes than the art of medicine. After enumerating the many benefits resulting from an application of steam and electricity, he said that Doctor Jenner's wonderful discovery of vaccination was of far greater importance to the world, and he felt certain that his audience would thoroughly agree with this idea. He reviewed the advancement made in medicine and its various branches, although it was a fact, he said, that republics rendered very little encouragement or aid to this art, as contrasted with monarchies, and consequently the Vanderbilts deserved all the more praise for their noble aid to the cause of medicine in America. The speaker also referred to Mrs. Wm. D. Sloane's large-hearted generosity, and at the end of the address Dr. Hall pronounced the benediction, and the audience made an inspection of the buildings. The physicians in charge of these two institutions are nearly all members of the faculty of the College of Physicians and Surgeons. Dr. James W. McLane, who is the family physician of the Vanderbilts, will be the attending physician to the hospital, and Dr. James W. Markoe, a son of the prominent surgeon, will be the house physician. The consultants, assistants and clinical staff are most all eminent men in the college faculty. As all the beds in the hospital have been endowed by Mrs. Sloane they will always be free.

At a session of the Pathological Society, Dr. J. Lewis Smith exhibited specimens, showing septic peritonitis and endocarditis, taken from the body of a new-born child. The mother's labor was normal and the infant, a female, appeared well till a week after birth, when restlessness, anorexia and fever came on. The breathing became hurried with moaning expirations. The temperature rose and was 104° F. in two days. Other symptoms were vomiting and some twitchings of left extremities. Upon examination of the chest no disease was discovered, but the abdomen was prominent and a probable diagnosis of septic peritonitis was made. Death occurred on the third day of the disease. The autopsy revealed a moderate amount of sero-pus in the abdominal cavity and a coating over all the abdominal viscera of fibrin, with some matting of the intestines. The umbilical vein was found pervious and contained pus and blood-clots, but the umbilicus had healed. There was also thickening of the aortic valve, a small thrombus being attached to one of the segments. A microscopic examination showed no signs of inflammation in the umbilical vein, but it was thought that it contained septic material which caused the peritonitis and endocarditis. Septicæmia of the new-born was said to be usually due to putrefying matter in the umbilical vein, accompanied with a rapid growth of bacteria. This decomposed material reaches the lines through branches of the umbilical vein and passes through the ductus venosus into the inferior vena cava. Having reached the general circulation, this septic matter may produce inflammation either upon the surface of the organs, or only infect the interior portions. In the way of treatment it was said that little could be accomplished, as this disease was one of the most fatal seen in infancy, but thorough cleanliness as a preventive was very essential. To prevent the entrance of bacteria through the umbilicus into the vein, the water, sponges and bandages used should be perfectly clean, and some antiseptic powder, as iodoform, dusted over the umbilicus was recommended. The President, Dr. T. Mitchell Prudden, said that in many cases of acute, ulcerative endocarditis micro-organisms, were found which produced phlebitis and also peritonitis. He considered that in the case reported there was in all probability a relation between the inflammation of the umbilical vein and the peritonitis and endocarditis. The outward appearance of the umbilicus, he remarked, did not indicate the absence of phlebitis.

For the purpose of discouraging all extravagant display at funerals, we have now an organization called the Burial Reform Association. This society met recently for the purpose of adopting a set of rules and electing officers. It is intended that many of the customs associated with modern funerals shall be done away with, as the use of crape and similar signs of mourning, flowers and numerous carriages. Among the medical men who are interested in this work may be mentioned Dr. Andrew H. Smith, Dr. D. B. St. John Rosa and others, and as the object is a very commendable one, a successful future may be looked for.

The New York Cremation Society recently held its annual meeting and reported progress. It was decided to co-operate with the Burial Reform Association and reduce the charges to $25, including all funeral expenses. A. F.

January, 1888.

